# Supplementary material for: Augmentation of myocardial If dysregulates calcium homeostasis and causes adverse cardiac remodeling
Source: Nat Commun. 2019 Jul 23;10:3295. doi: 10.1038/s41467-019-11261-2 (PMC6650438; doi:10.1038/s41467-019-11261-2)
Supplement: Supplementary file 1 — Supplementary Information [file 41467_2019_11261_MOESM1_ESM.pdf]

Supplementary Information

Augmentation of myocardial I_f dysregulates calcium homeostasis and causes adverse cardiac remodeling

Yampolsky et al.

a b

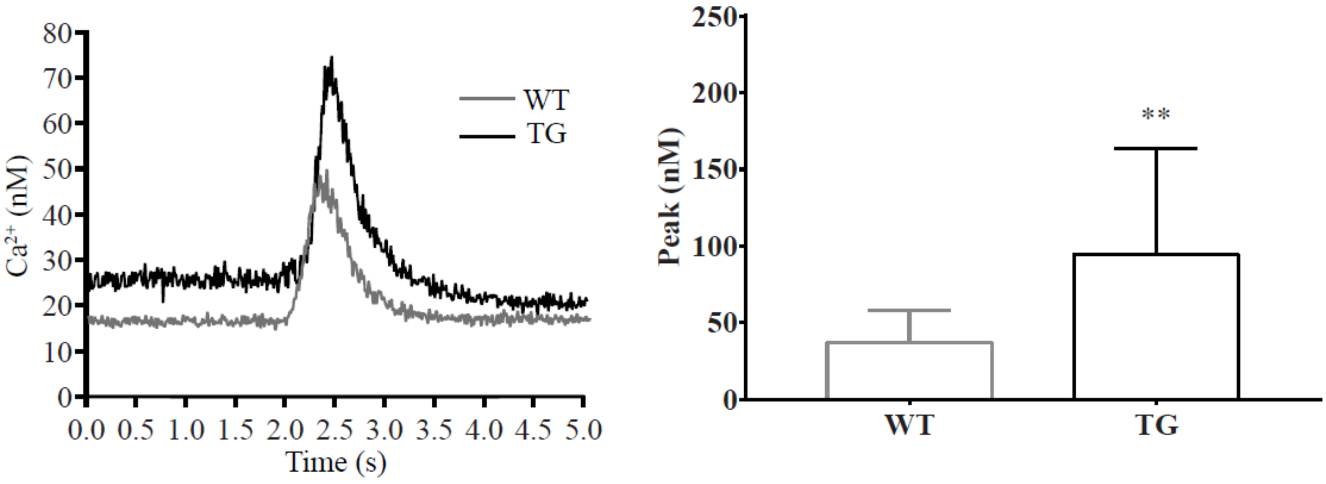

Supplementary Figure 1

Experiments were performed in isolated cardiomyocyte preparation (load with 10 µM Fluo4-AM, puff with caffeine 10 mM). Quantification of the amplitude [peak] of caffeine-induced Ca^{2+} transients, which serves as a measure for sarcoplasmic reticulum Ca^{2+} content. **a**, representative Ca^{2+} transients. **b**, results of the experiment (transgenic $HCN4^{tg/wt}$ cells = TG [n=20]; wild type cells = WT [n=12]; mean ± standard deviation; **P<0.01 TG vs. WT; two-tailed Mann-Whitney test). Source data are provided as a Source data file.

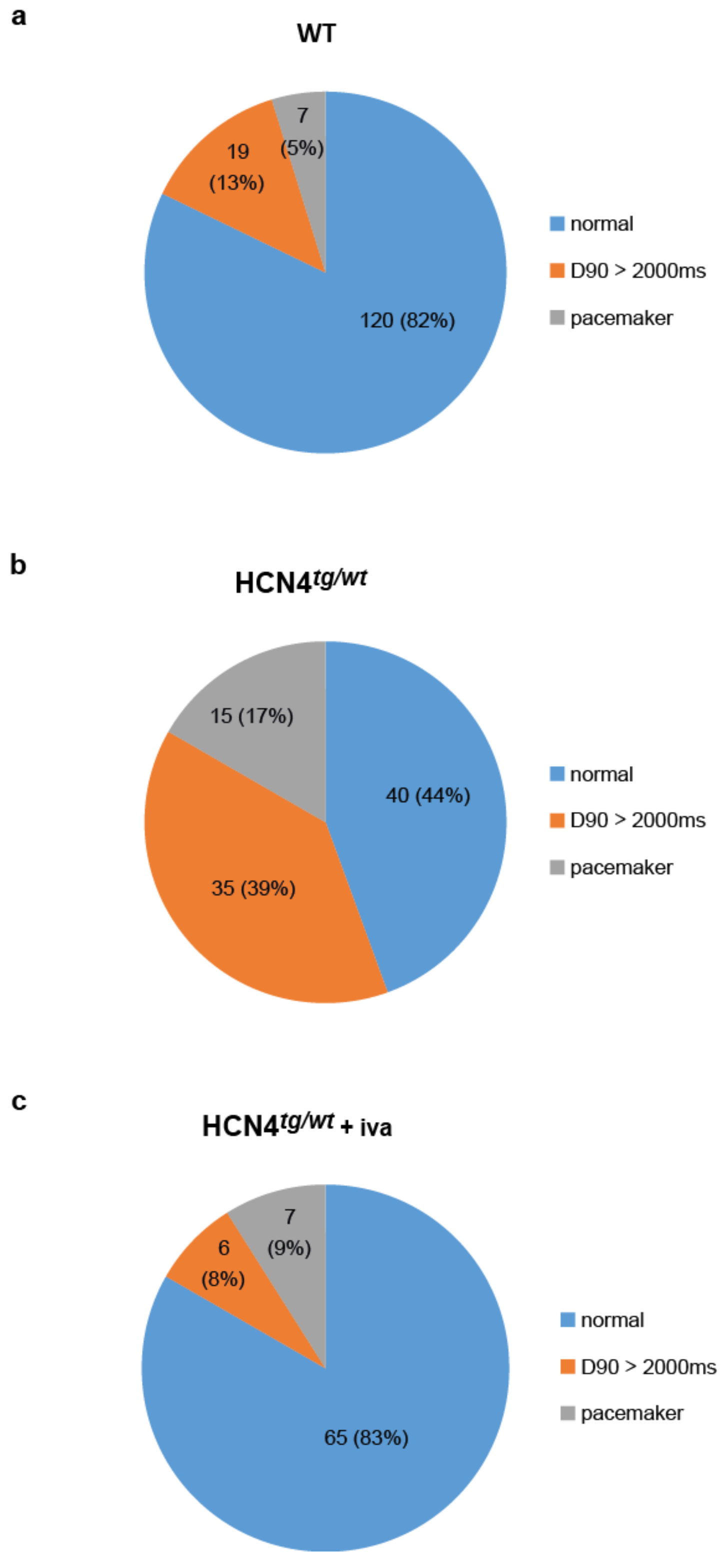

Supplementary Figure 2

Properties of Ca^{2+} transients recorded from isolated cardiomyocytes of wild type and *HCN4$^{tg/wt}$* hearts. a**,** cardiomyocytes isolated from wild type mice predominantly showed normal behaviour, lacking automaticity (defined as spontaneous beating rate >0.2 Hz) or prolonged decay of diastolic $[Ca^{2+}]_i$ (defined as D_{90} > 2000ms). **b,** In contrast, considerable numbers of cardiomyocytes from *HCN4$^{tg/wt}$* mice revealed spontaneous pacemaker activity, characterized by periodic firing of $[Ca^{2+}]_i$ transients, or $[Ca^{2+}]_i$ transients with abnormal prolongation of diastolic $[Ca^{2+}]_i$ decay (defined as D_{90} > 2000ms). **c,** These changes recovered to the wild type situation when *HCN4$^{tg/wt}$* cardiomyocytes were pre-treated with ivabradine (3 µM). Notably, only $[Ca^{2+}]_i$ transients with normal configuration were used for the evaluation according to Figures 5 and 6 of the main manuscript.

a

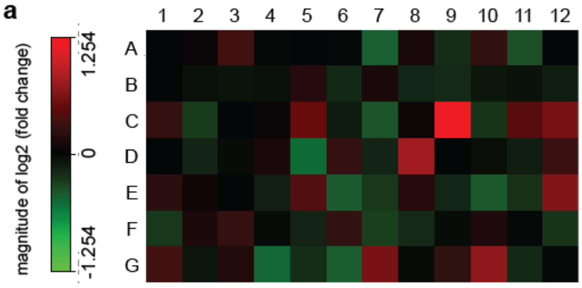

b

	1	2	3	4	5	6	7	8	9	10	11	12
A	Akt1 1.00	Apaf1 1.02	Api5 1.27	Atf5 -1.01	Bad 1.00	Bag1 -1.00	Bag3 -1.38	Bak1 1.08	Bax -1.16	Bcl10 1.20	Bcl2 -1.31	Bcl2l1 1.00
B	Bcl2l10 1.00	Bcl2l2 -1.04	Bid -1.05	Naip1 -1.04	Naip2 1.14	Birc2 -1.14	Birc3 1.08	Xiap -1.13	Birc5 -1.15	Bnip2 -1.06	Bnip3 -1.04	Bnip3l -1.09
C	Bok 1.22	Card10 -1.22	Nod1 1.00	Card6 1.02	Casp1 1.43	Casp12 -1.09	Casp14 -1.33	Casp2 1.03	Casp3 2.39	Casp4 -1.19	Casp6 1.36	Casp7 1.51
D	Casp8 1.00	Casp9 -1.11	Cflar -1.02	Cidea 1.09	Cideb -1.44	Cradd 1.21	Dad1 -1.11	Dapk1 1.73	Dffa -1.00	Dffb -1.03	Tsc22d3 -1.09	Fadd 1.25
E	Fas 1.16	Fasl 1.05	Hells 1.00	Il10 -1.10	Lhx4 1.34	Ltbr -1.36	Mcl1 -1.21	Nfkb1 1.14	Nme5 -1.13	Nol3 -1.35	Pak7 -1.18	Pim2 1.57
F	Polb -1.21	Prdx2 1.09	Pycard 1.22	Ripk1 -1.02	Rnf7 -1.12	Sphk2 1.20	Tnf -1.24	Tnfrsf10b -1.15	Tnfrsf11b -1.02	Tnfrsf1a 1.10	Cd40 -1.01	Tnfsf10 -1.20
G	Tnfsf12 1.27	Cd40lg -1.06	Cd70 1.11	Traf1 -1.42	Traf2 -1.16	Traf3 -1.37	Trp53 1.51	Trp53bp2 -1.02	Trp53inp1 1.18	Trp63 1.63	Trp73 -1.14	Zc3hc1 -1.01

Supplementary Figure 3

Mouse apoptosis array. a, b. Heat map representation (a) and numerical data (b) of apoptosis related gene expression in *HCN4*$^{tg/wt}$ as fold-change of wild type values (wt = 1.0). 96-well positions are indicated with expression levels colour-coded in black (no change), red (increased level) and green (decreased level).

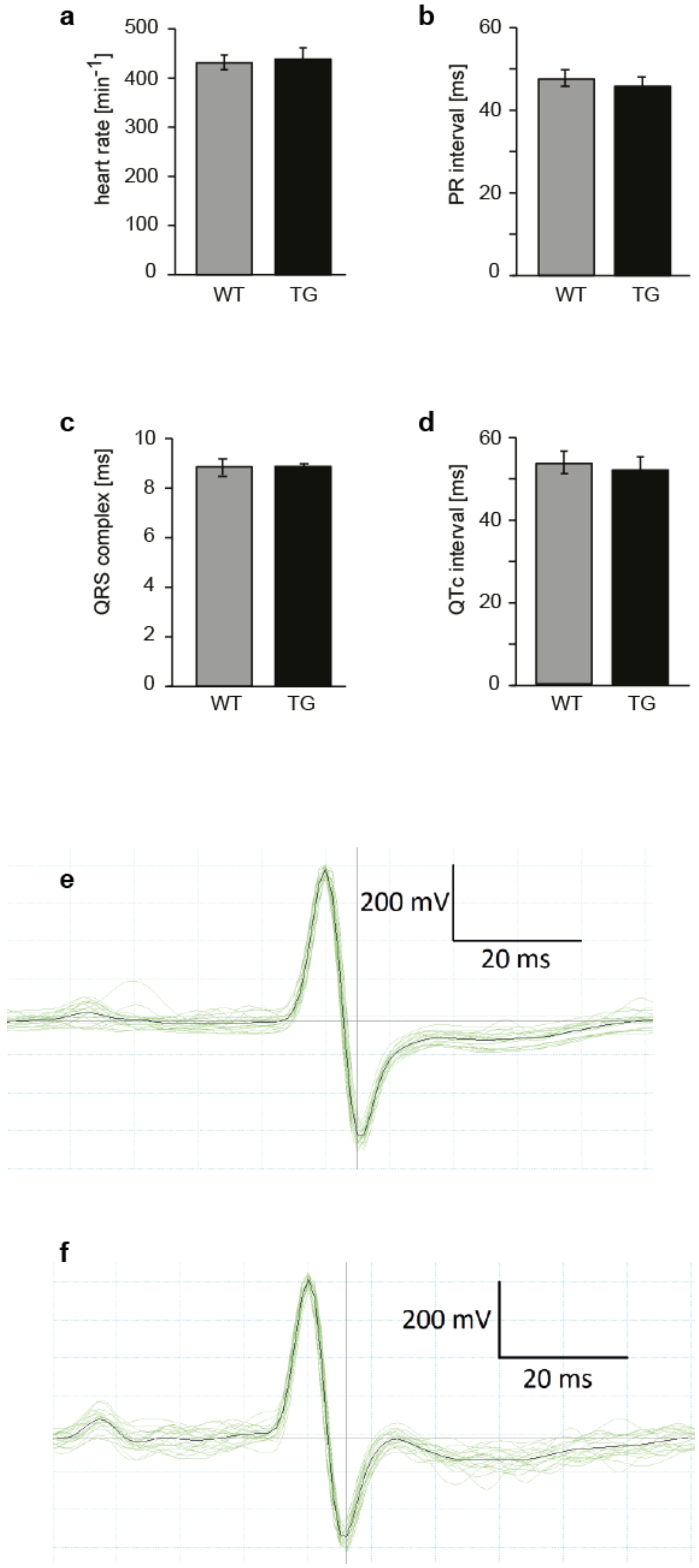

Supplementary Figure 4

Key surface ECG parameters in anaesthetized mice (3 months of age). a-d. heart rate (a). PR interval (b), QRS complex (c) and QTc interval (d) measured in 3 months-old wild type (WT, grey columns, n= 23 animals) and *HCN4*$^{tg/wt}$ (TG, black columns, n = 6 animals) mice. Data are presented as mean ± s.e.m. * P<0.05, ** P<0.01, *** P<0.001; unpaired t-test. **e, f** Representative ECG complexes as overlay of 50 consecutive complexes from wild type (e) and transgenic (f) mice. Source data are provided as a Source data file.

Figure 1c

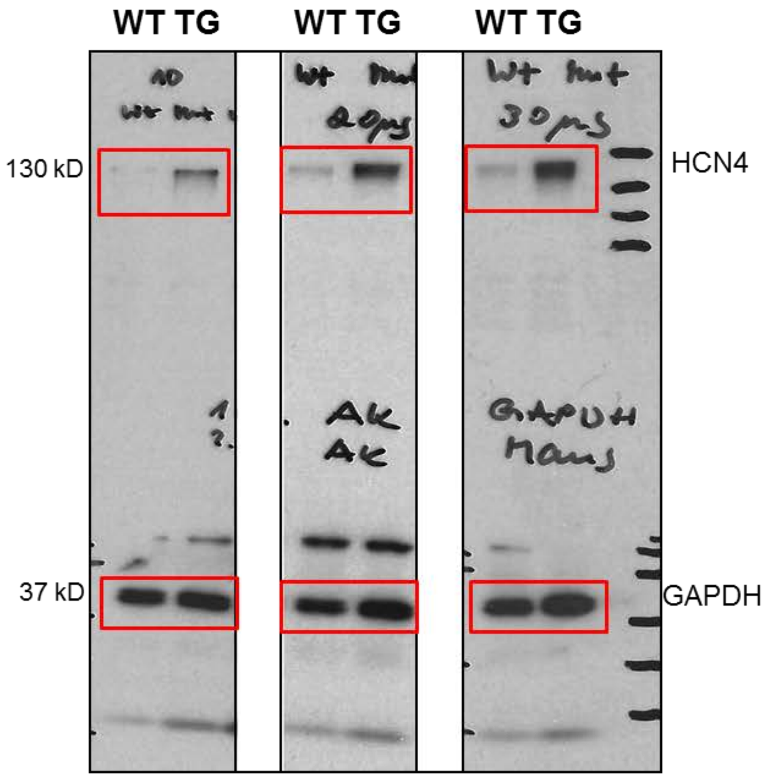

Figure 4f

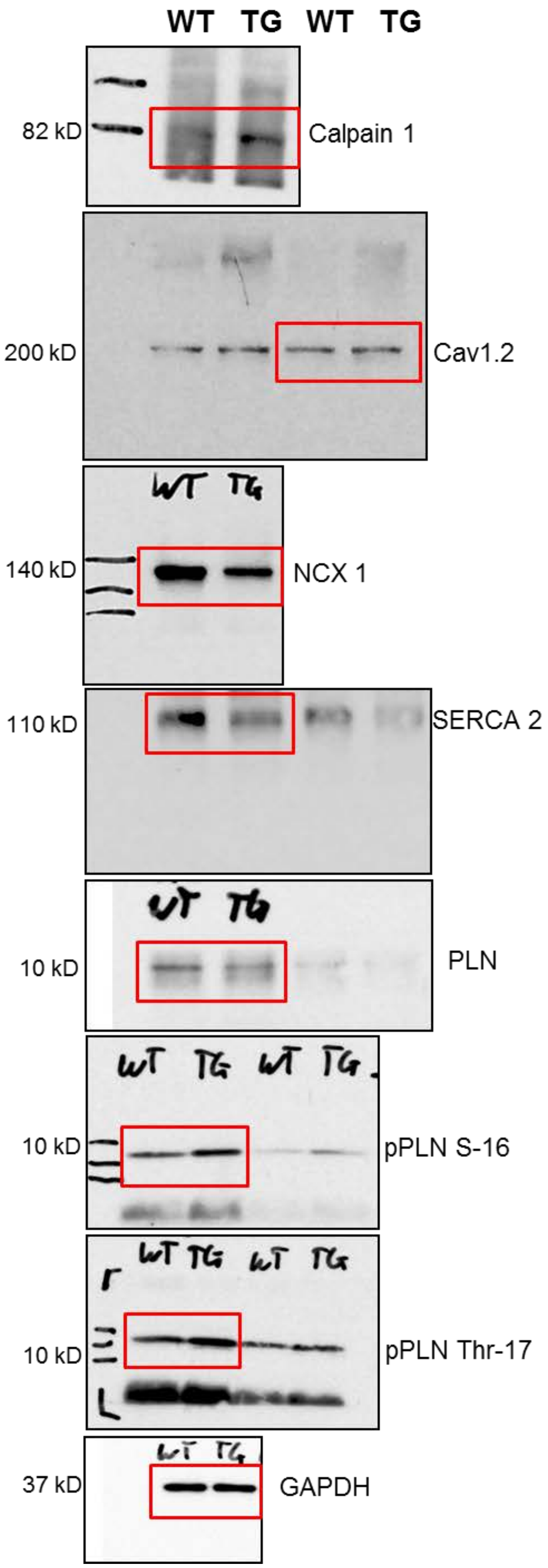

Supplementary Figure 5

Western blot images used to compose result panels in indicated figures.

	WT	*HCN4* $^{+/tg}$	*P value*
N	5	5	
RR Interval (ms)	107.3 ± 5.5	106.7 ± 11.2	0.92
Heart Rate (BPM)	569.4 ± 27.1	576.9 ± 52.8	0.79
PR Interval (ms)	37.2 ± 1.9	35.5 ± 1.3	0.14
P Duration (ms)	14.4 ± 1.3	13.7 ± 1.4	0.44
QRS interval (ms)	16.3 ± 0.9	16.1 ± 1.6	0.82
QTc (ms)	46.9 ± 3.1	50.8 ± 2.1	0.053
PVCs / 10^4 beats	1.6 ± 1.1	18.4 ± 5.5	0.018
Mice with nonsustained VT	0	3	0,1667

Abbreviations and definitions: BPM beats per minute, PVCs premature ventricular captures, VT ventricular tachycardia. Nonsustained VT, ≥ 3 consecutive ventricular beats.

Supplementary Table 1

Ambulatory telemetry of wild type (WT) and transgenic (*HCN4* $^{+/tg}$) mice (6 months of age). Data are presented as mean ± s.e.m. Source data are provided as a Source data file.

Human target gene	Human target protein	Primer accession number (TaqMan assay)
GAPDH	GAPDH	Mm99999915_m1
HPRT1	HPRT1	Mm00446968_m1
ACTB	ß-actin	Mm00607939_m1
SCN5A	Na_v1.5	Mm00451971_m1
SLC8A1	NCX	Mm01232254_m1
Ryr2	RyR2	Mm00465877_m1
CACNA1C	Ca_v1.2	Mm00437917_m1
CACNA1D	Ca_v1.3	Mm01209919_m1
CACNA1A	Ca_v2.1	Mm_00432190_m1
CACNA1G	Ca_v3.1	Mm00486572_m1
KCNJ2	K_{ir}2.1	Mm00434616_m1
KCNA4	K_v1.4	Mm00445241_s1
KCNA5	K_v1.5	Mm00524346_s1
KCND2	K_v4.2	Mm01161732_m1
KCND3	K_v4.3	Mm01302126_m1
KCNQ1	K_v7.1	Mm00434640_m1
KCNH2	hERG	Mm00465370_m1
KCNIP2	KChip2	Mm00518915_g1
mHCN1	mHCN1	Mm00468832_m1
mHCN2	mHCN2	Mm00468538_m1
mHCN4	mHCN4	Mm01176086_m1
hHCN4	hHCN4	Hs00175760_m1
Myh7b	Myosin heavy chain 7B	Mm01249941_m1
ATP2a2	Serca2a	Mm01201431_m1
Pln	Pln	Mm04206541_m1
Ppp3ca	Caln	Mm01317678_m1
Atp1a1	Na^+/K^+-ATPase	Mm00523255_m1
Nppa	ANP	Mm01255747_g1
Casp3	Casp3	Mm01195085_m1
Tgm2	tTG	Mm00436987_m1
Capn1	Capn1	Mm00482964_m1
Mtor	mTOR	Mm00444968_m1
Gsk3b	GSK-3B	Mm00444911_m1

Supplementary Table 2

Primers used for quantitative real-time polymerase chain reaction (qRT-PCR).

Primary antibody	Secondary antibody
Anti-NCX1 antibody ab135735 (IgG, rabbit, abcam)	Anti-Rabbit IgG (whole molecule) Peroxidase (A6154) (Sigma-Aldrich)
Anti-Ca_v1.2 (#ACC-003) (IgG, rabbit, Alomone Labs)	Anti-Rabbit IgG (whole molecule) Peroxidase (A6154) (Sigma-Aldrich)
Anti-Serca2 (F-1): sc-376235 (IgG, mouse, Santa Cruz Biotechnology)	Goat anti-mouse IgG-HRP: Sc-2005 (Santa Cruz Biotechnology)
Anti-Phospholamban antibody [2D12] (ab2865) (IgG, mouse, abcam)	Goat anti-mouse IgG-HRP: Sc-2005 (Santa Cruz Biotechnology)
A010-12 Anti-phospho-PLB (Ser-16) (IgG, rabbit, Badrilla)	Anti-Rabbit IgG (whole molecule) Peroxidase (A6154) (Sigma-Aldrich)
A010-13 anti-phospho-PLB (Thr-17) (IgG, rabbit, Badrilla)	Anti-Rabbit IgG (whole molecule) Peroxidase (A6154) (Sigma-Aldrich)
Anti-Calpain 1 antibody (ab28258) (IgG, rabbit, abcam)	Anti-Rabbit IgG (whole molecule) Peroxidase (A6154) (Sigma-Aldrich)
Anti-HCN4 (#APC-052) (IgG, rabbit, Alomone Labs)	Anti-Rabbit IgG (H+L), F(ab) fragment-Peroxidase antibody (Sigma-Aldrich)
GAPDH (HRP) G8140-11 Mab (IgG, mouse, Us Biologicals)	Goat anti-mouse IgG-HRP: Sc-2005 (Santa Cruz Biotechnology)

Supplementary Table 3

Antibodies used for immunocytochemical assays.
