# Supplementary material for: Augmentation of myocardial If dysregulates calcium homeostasis and causes adverse cardiac remodeling
Source: Nat Commun. 2019 Jul 23;10:3295. doi: 10.1038/s41467-019-11261-2 (PMC6650438; doi:10.1038/s41467-019-11261-2)
Supplement: Supplementary file 3 — Reporting Summary [file 41467_2019_11261_MOESM3_ESM.pdf]

nature research

Corresponding author(s): Patrick A. Schweizer

Last updated by author(s): May 25, 2019

Reporting Summary

Nature Research wishes to improve the reproducibility of the work that we publish. This form provides structure for consistency and transparency in reporting. For further information on Nature Research policies, see Authors & Referees and the Editorial Policy Checklist.

Statistics

For all statistical analyses, confirm that the following items are present in the figure legend, table legend, main text, or Methods section.

n/a	Confirmed	
☐	☒	The exact sample size (*n*) for each experimental group/condition, given as a discrete number and unit of measurement
☐	☒	A statement on whether measurements were taken from distinct samples or whether the same sample was measured repeatedly
☐	☒	The statistical test(s) used AND whether they are one- or two-sided<br>*Only common tests should be described solely by name; describe more complex techniques in the Methods section.*
☒	☐	A description of all covariates tested
☐	☒	A description of any assumptions or corrections, such as tests of normality and adjustment for multiple comparisons
☐	☒	A full description of the statistical parameters including central tendency (e.g. means) or other basic estimates (e.g. regression coefficient) AND variation (e.g. standard deviation) or associated estimates of uncertainty (e.g. confidence intervals)
☒	☐	For null hypothesis testing, the test statistic (e.g. *F*, *t*, *r*) with confidence intervals, effect sizes, degrees of freedom and *P* value noted<br>*Give P values as exact values whenever suitable.*
☒	☐	For Bayesian analysis, information on the choice of priors and Markov chain Monte Carlo settings
☒	☐	For hierarchical and complex designs, identification of the appropriate level for tests and full reporting of outcomes
☒	☐	Estimates of effect sizes (e.g. Cohen's *d*, Pearson's *r*), indicating how they were calculated

Our web collection on statistics for biologists contains articles on many of the points above.

Software and code

Policy information about availability of computer code

Data collection	Bright-field images were digitally recorded on Axioplan2 microscope (Zeiss, Oberkochen, Germany) with an attached 3CCD camera (Intas, Goettingen, Germany). Immunofluorescent staining was imaged using a Leica confocal laser scanning unit TCS NT, which was coupled to a Leica DM IRB microscope. Acquisition of image series was performed using TCS NT (Leica, Heidelberg, Germany) software. For electron microscopy sections were viewed on Philips EM400T (Philips IS, Eindhoven, The Netherlands) and images were documented on Image Plates and scanned with the Micro Imaging Plate Scanner (DITABIS, Pforzheim, Germany). [Ca2+]i transients were recorded using an Olympus OSP-3 System fluorescence microscope. The fluorescence signal was A/D converted using PowerLab 4/35 and LabChart V7.0. If and AP recordings were done with an Axopatch 200B amplifier (Molecular Devices, Sunnyvale, USA), digitized at 20kHz with a 1401 Power3 Analog/Digital Converter (CED, Cambridge, UK) and stored on a hard drive. Quantitative real-time PCR (RT-qPCR) was performed using an ABS 7500 Realtime PCR System (Thermo Fisher, Waltham, MA, USA). The Vevo® 2100 device (VisualSonics, Toronto, Canada) with the corresponding transducer MS-400 was used to record the echocardiographies. For telemetric ECG recordings, a radiotelemetry transmitter (model EA-F20, Data Sciences International, St. Paul, Minnesota, USA) connected to a Powerlab System (AD Instruments, Hastings, UK) was used.
Data analysis	ECG recordings were analysed using the Chart 5 software (AD Instruments, Hastings, UK). Apoptosis related gene expression data were analysed using online software "RT2 Profiler PCR Array Data Analysis" (version 3.5) on the manufacturer's website http://pcrdataanalysis.sabiosciences.com/pcr/arrayanalysis.php. If and AP recordings were analysed off-line with custom MATLAB routines (Mathworks, Natick, USA) software. All images were processed in ImageJ version 1.47 (NIH, Bethesda, MD, USA) software using standard quantification methods and standard ImageJ plug-ins. Data analysis and statistical evaluation was undertaken using Excel 2010 (Microsoft, Redmond, WA, USA) and GraphPad Prism 5.0 (GraphPad Prism Inc. La Jolla, CA).

For manuscripts utilizing custom algorithms or software that are central to the research but not yet described in published literature, software must be made available to editors/reviewers. We strongly encourage code deposition in a community repository (e.g. GitHub). See the Nature Research guidelines for submitting code & software for further information.

Data

Policy information about availability of data

All manuscripts must include a data availability statement. This statement should provide the following information, where applicable:

- Accession codes, unique identifiers, or web links for publicly available datasets
- A list of figures that have associated raw data
- A description of any restrictions on data availability

The data that support the findings of this study are available from the article and the supplementary information files, or from the corresponding author upon reasonable request. A reporting summary for this article is available as a supplementary information file. Source data underlying the Figs. 1b and 1e, 2b-g and 2i-q, 3b and 3d, 4a-e, 5b-f, 6b-f, 7d and 7f, 8b-f, 9g, and Supplementary Figs. 1, 4, and Supplementary Table 1 are provided as a source data file.

Field-specific reporting

Please select the one below that is the best fit for your research. If you are not sure, read the appropriate sections before making your selection.

☒ Life sciences ☐ Behavioural & social sciences ☐ Ecological, evolutionary & environmental sciences

For a reference copy of the document with all sections, see nature.com/documents/nr-reporting-summary-flat.pdf

Life sciences study design

All studies must disclose on these points even when the disclosure is negative.

Sample size	Sample sizes were chosen based on previous experience with the animal model, following convention of the methods and considered to be sufficient based on publications in the field. No statistical methods were used to determine sample size.
Data exclusions	No data was excluded from the analyses.
Replication	Data described in this manuscript were reliably reproduced.
Randomization	Animals and cells were randomly assigned to the experimental groups.
Blinding	The investigators were blinded to group allocation during data collection and analysis.

Reporting for specific materials, systems and methods

We require information from authors about some types of materials, experimental systems and methods used in many studies. Here, indicate whether each material, system or method listed is relevant to your study. If you are not sure if a list item applies to your research, read the appropriate section before selecting a response.

Materials & experimental systems

n/a	Involved in the study	
☐	☒	Antibodies
☒	☐	Eukaryotic cell lines
☒	☐	Palaeontology
☐	☒	Animals and other organisms
☒	☐	Human research participants
☒	☐	Clinical data

Methods

n/a	Involved in the study	
☒	☐	ChIP-seq
☒	☐	Flow cytometry
☒	☐	MRI-based neuroimaging

Antibodies

Antibodies used	Described in the Methods section, details uploaded as Supplementary Table 3
Validation	Validations are based on the datasheets from the manufacturers. Only previously validated, commercial antibodies were used. Optimization was performed by testing a range of dilutions of each antibody.

Animals and other organisms

Policy information about studies involving animals; ARRIVE guidelines recommended for reporting animal research

Laboratory animals	2, 3, and 6 months-old male C57BL/6N mice were used for the experiments.

Wild animals	No wild animals were used.
Field-collected samples	No field-collected samples were used.
Ethics oversight	All experiments were carried out in accordance with the Guide for the Care and Use of Laboratory Animals published by the US National Institute of Health (NIH publication number 85–23, revised 1996), and with the European Community guidelines for the use of experimental animals. Protocols were approved by the local regulatory authority (#35-9185.81/G-20/11 and #35-9185.81/G-226/16 Regierungspräsidium Karlsruhe, Germany).

Note that full information on the approval of the study protocol must also be provided in the manuscript.
